# Progress in Surgery of the Stomach

**Published:** 1901-08-10

**Authors:** 


### Progress in Surgery of the Stomach.

(*Continued from page* 301.)

**Ulcer of the Stomach.**—Robson[8] enumerates the complications of this affection as follows:—(1) Local peritonitis or perigastritis ending in adhesions. (2) Local peritonitis ending in suppuration and a localised abscess. (3) Subphrenic abscess. (4) Abscess of the liver, pancreas, or spleen. (5) Fistula between the stomach or pylorus and adjoining organs or with the surface of the body. (6) Acute perforation of the stomach wall. (7) General peritonitis. (8) Hæmatemesis and melæna. (9) Dilatation of the stomach. (10) Tumour of the stomach or the pylorus. (11) Cicatricial stenosis of the pylorus. (12) Hour-glass stomach. (13) Spasm of the pylorus, producing intermittent narrowing (Reichmann's disease). (14) Atonic motor deficiency. (15) Severe gastralgia. (16) Persistent vomiting. (17) Tetany. (18) Acute or chronic pancreatitis. (19) Profound anæmia, resembling the pernicious form. (20) Pressure on, or stricture of, the bile ducts with jaundice. (21) Catarrh of the gall bladder from adhesions, producing attacks like those of cholelithiasis. (22) Great loss of flesh and strength ending in phthisis. (23) Cancer secondary to ulcer ("Ulcus carcinomatosum"). Bidwell[9] publishes two cases of pyloroplasty for cicatricial contraction of the pylorus following gastric ulcer, and cases of operation for perforated gastric ulcer are reported by M'Laren,[10] Atherton,[11] Rice,[12], and Mackay.[13] Robson[14] urges the following points in the treatment of gastric ulcer: (1) All cases of acute uncomplicated gastric ulcer should be submitted to thorough medical treatment in the shape of long-continued rest and attention to diet, the patients not being allowed to get up or resume solid food until at least a fortnight after all disappearance of pain. (2) Where the ulceration persists and proves intractable to medical treatment, or where relapses occur, gastro-enterostomy should be performed so as to secure physiological rest and relieve the hyperacidity of the gastric juice nearly always found in such cases. (3) Perforation demands immediate surgical treatment. (4) The complications of disabling adhesions around the stomach and pylorus, pyloric contraction, and hour-glass contraction, due to chronic ulcers leading to pain, dilatation, loss of flesh, and general impairment of health, now often treated as chronic indigestion, should be treated surgically. (5) In recurring or so-called chronic hæmatemesis from gastric ulcers surgical treatment is decidedly called for. (6) In acute hæmatemesis further accuracy in diagnosis as to the size of the bleeding vessels is urgently needed; and the co-operation of the physician and surgeon is advisable in all cases of hæmatemesis, so that if relief be not obtained by medical and general treatment surgical means may be adopted if the bleeding is believed to occur from a large vessel; but seeing that capillary hæmorrhage is capable of relief by medical means alone, medical should always precede surgical treatment. Rodman[15] discusses the treatment of cases of gastric ulcer not amenable to medical treatment in a reasonable time, in which there is severe pain and uncontrollable vomiting. The operation of excision, having a mortality of 15 per cent., cannot be advised in all cases, but should be held in reserve for the more severe ones. If the ulcer is at the pylorus, or upon the anterior wall, being therefore accessible and easily removable, pylorectomy is preferable to gastro-enterostomy; but if it be situated posteriorly, and at the same time adherent, as it frequently is, to adjacent structures, then gastro-enterostomy is preferable. If the ulcers are multiple, gastro-enterostomy should be employed. When a case has bled freely once, has recovered, and then in a few days has bled seriously again, pylorectomy if the ulcer is at the pylorus, or gastrectomy for ulcers situated elsewhere, is the ideal operation. Both the hæmorrhage and the risk of perforation are cogent reasons for the complete operation should the conditions be favourable. Ligation of the bleeding vessel in situ is not to be recommended, and suture of the ulcer, cauterisation, pyloroplasty, gastrorrhaphy, and curetting of the ulcer with or without cauterisation are not to be preferred to the methods advised. Rydygier[16] strongly advocates radical resection of the pylorus in cases of gastric ulcer or cicatricial puckering after ulceration, provided, of course, that no contra-indication is given by the general condition, for the following reasons:—1. The ulcer or cicatrix may, he believes, lead to the development of carcinoma. 2. In spite of a gastro-enterostomy previously performed, fatal hæmorrhage may occur. 3. After a gastro-enterostomy it is not uncommon for a "circulus vitiosus" of the intestinal ingesta to be established. Moullin[17] records three cases of gastrotomy for hæmatemesis, and Chapman[18] is quoted to show that profuse hæmatemesis and symptoms of perforation can occur without discoverable lesion at the autopsy.

[8] Lancet, May 25, 1901, p. 1454, and Brit. Med. Jour., Feb. 2, 1901, p. 257. [9] Brit. Med. Jour., Oct. 6, 1900, p. 1000. [10] Scottish Med. Journ., Oct. 1900, p. 319. [11] Med. Rec., Jan. 5, 1901, p. 12. [12] Lancet, Jan. 26, 1901, p. 248. [13] Lancet, Feb. 16, 1901, p. 470. [14] The Medical Press, Feb. 13, 1901, p. 161. [15] Brit. Med. Jour., Oct. 27, 1900, Epit. No. 185. [16] Brit. Med. Jour., Jan. 19, 1901, Epit. No. 45. [17] Lancet, Oct. 20, 1900, p. 1125. [18] Lancet, April 27, 1901, p. 1220.

